# Marginal Quality and Wear of Bulk-fill Materials for Class-II Restorations in Primary Molars

**DOI:** 10.3290/j.jad.b4051483

**Published:** 2023-04-25

**Authors:** Maria Hofmann, Stefanie Amend, Susanne Lücker, Roland Frankenberger, Bernd Wöstmann, Norbert Krämer

**Affiliations:** a Asisstant Professor, Department of Pediatric Dentistry, Medical Center for Dentistry, University Medical Center Giessen and Marburg, Campus Giessen, Giessen, Germany. Performed the experiments, wrote the manuscript.; b Asisstant Professor, Department of Paediatric Dentistry, Medical Center for Dentistry, University Medical Center Giessen and Marburg, Campus Giessen, Giessen, Germany. Data analysis.; c Research Fellow, Department of Paediatric Dentistry, Medical Center for Dentistry, University Medical Center Giessen and Marburg, Campus Giessen, Giessen, Germany. Supervised experiments.; d Professor and Chair, Department of Operative Dentistry, Endodontics, and Pediatric Dentistry, University of Marburg, Medical Center for Dentistry, University Medical Center Giessen and Marburg, Campus Marburg, Marburg, Germany. Study concept, proofread the manuscript.; e Professor and Chair, Department of Prosthodontics, Medical Center for Dentistry, University Medical Center Giessen and Marburg, Campus Giessen, Giessen, Germany. Supervised thermomechanical loading and wear analysis.; f Professor and Chair, Department of Pediatric Dentistry, Medical Center for Dentistry, University Medical Center Giessen and Marburg, Campus Giessen, Giessen, Germany. Study idea, supervised the experiments, proofread the manuscript.

**Keywords:** Class-II restoration, composite, marginal analysis in SEM, polyacid modified resin, wear.

## Abstract

**Purpose:**

The aim of this in-vitro study was to evaluate the marginal integrity and wear of eight bulk-fill materials in comparison to a compomer in Class-II cavities in primary molars after thermomechanical loading (TML).

**Materials and Methods:**

Prepared Class-II cavities in 72 extracted primary molars were filled with eight bulk-fill materials. A compomer served as the control group. After water storage (incubator, 28 days, 37°C), samples were subjected to TML (2500 thermal cycles 5°C/55°C; 100,000 load cycles, 50 N, 1.67 Hz). Before and after TML, replicas were made which were used for both SEM analysis of marginal integrity and 3-D wear analysis. Statistical analysis was performed using Kruskal-Wallis and Wilcoxon tests (p < 0.05).

**Results:**

A significant reduction in perfect margins was observed for all groups, while marginal gap formation increased (Wilcoxon test, p < 0.02) for all groups but the compomer. Significant interindividual differences were observed between the tested materials regarding marginal integrity (Kruskal-Wallis test, p < 0.05). Wear analysis revealed no significant differences between groups (Kruskal-Wallis test, p > 0.05).

**Conclusion:**

Some of the bulk-fill materials investigated here achieved better results than the compomer and should be further evaluated clinically.

Since the signing of the Minamata Convention on Mercury,^7^ countries worldwide have reduced the use of dental amalgam, especially in pediatric dentistry,^4^ while materials science has continued to evolve. Although tooth-colored restorative materials are primarily used today in restorative treatment in the primary dentition,^27^ dental amalgam is still recommended with strong clinical evidence for the restoration of primary molars.^3^ Nevertheless, sufficient evidence is lacking for the one preferable tooth-colored material for restoration of Class-II cavities in primary molars,^15,35,39^ but resin-based materials seem to be preferable with regard to their clinical performance.^30,39^ Bulk-fill composite resins (BFC) have been developed to reduce operation time by allowing simple, quick application of the filling material,^5,31,38^ which can be advantageous in pediatric dentistry. With BFC, depths of cure of 4-5 mm can be achieved^17^ which makes it possible to apply the filling material in one single layer, thus avoiding an incremental technique. For the permanent dentition, BFC achieves comparable and in some cases even better results compared to conventional composite resins in vitro and in vivo with follow-up periods of up to ten years.^6,20,43^ Concerning the use of BFC in primary molars, there are only a few clinical trials with follow-up periods of up to two years.^2,10,31,38^

Based on their physical and chemical properties, BFC can be divided into two groups: high-viscosity (moldable) and low-viscosity (flowable) BFC.^17^ The viscosity of the filling material as well as polymerization shrinkage affect the formation of microleakage between dental hard tissues and filling materials.^34^ When microleakage allows the passage of bacteria, secondary caries can occur.^1^ These carious lesions at the restoration margins are one of the main reasons for failure of dental restorations in permanent and primary teeth^8,26^ and, when untreated, can result in (irreversible) infection of the pulp.^1^ Class-II fillings, that is, especially their gingival restoration margins, and patients with a high individual caries risk are particularly susceptible to secondary caries.^29^ In high stress-bearing areas such as posterior teeth, wear resistance of filling materials is also of particular concern;^41^ however, due to the short lifetime of deciduous teeth, this problem is less important than marginal quality.

The aim of this in-vitro study was to investigate whether marginal integrity and wear of bulk-fill materials for the restoration of Class-II cavities in the primary dentition are comparable to tooth-colored, polyacid-modified resin composites (compomers) which are frequently used in deciduous teeth and have been described as an adequate amalgam alternative in the primary dentition.^3,18^ The null hypothesis tested was that there would be no difference between the different types of materials investigated.

## Materials and Methods

Seventy-two primary molars, extracted for therapeutic reasons, were collected and stored in 0.5% chloramine-T for a maximum of 28 days, after which they were kept frozen. Verbal consent of patients and patient’s parents, as approved by the ethics committee of the Justus Liebig University, Giessen (file reference 143/09), was obtained. The inclusion and exclusion criteria for tooth selection are listed in Table 1. Teeth were randomly assigned to nine groups (n = 8).

**Table 1:** Inclusion and exclusion criteria for tooth selection

Inclusion criteria	Exclusion criteria
One caries-free proximal surface or After caries excavation: caries-free dentin surface (residual dentin thickness ≥ 1 mm) Caries-free enamel margins	After caries excavation (both proximal surfaces): pulp exposure or Residual dentin thickness < 1 mm
At least 1 tooth root (length ≥ 3 mm)	Fillings (both proximal surfaces)
	Enamel or dentin formation disorders

Prior to cavity preparation, teeth were cleaned with scalers and an air-polishing system (Prophypearls, KaVo; Biberach, Germany). Standardized mesial box-only Class-II cavities (3.0-3.5 mm bucco-oral width, 1.5-2.0 mm axial depth, 2.0-3.0 mm gingival depth; cervical margin above the cementoenamel junction) were prepared. With the exception of group 8, which was prepared with a self-adhesive material, all cavities were bonded with universal adhesives used in self-etch mode and combined with restorative materials from the same manufacturer. Seven BFC, one bulk-fill self-adhesive composite hybrid, and one compomer (control), were used as filling materials. The adhesives and restoratives along with their composition and application protocol are shown in Table 2. Bulk-fill materials were applied in one single layer, and the compomer was applied in three oblique layers with a maximum thickness of 2 mm. Application of the adhesive and every layer of filling material was followed by 30-s light curing (Bluephase G2, Ivoclar Vivadent; Schaan, Liechtenstein) with a light intensity of 1200 mW/cm^2^. Afterwards, restorations were finished and polished by one specific operator using 3M Sof-Lex polishing disks (3M Oral Care; St Paul, MN, USA) and Identoflex Composite Polisher (Kerr; Orange, CA, USA) at 5200 rpm. The quality of finishing and polishing was examined with a magnifying glass lamp (Magnifer GlassLamp, Modelnummer 8093, MBFZ toolcraft; Georgensmünd, Germany) and a reflected-light microscope (Nikon AZ100M in combination with Nikon DS-Ri1, both Nikon Europe BV; Amsterdam, Netherlands). The procedure was repeated until no more overhang was detectable under the magnifying glass lamp and the microscope.

**Table 2:** Materials investigated

Group	Brand name, abbreviation and manufacturer of filling material (FM)	FM color	FM composition	FM viscosity (during application process)	FM filler load	FM filler size	Brand name of adhesive and manufacturer	Adhesive composition
1	SDR flow+ (SDR, Dentsply DeTrey; Konstanz, Germany)	Universal shade	Barium-alumino-fluoro-borosilicate glass, strontium alumino-fluoro-silicate glass, ytterbium trifluoride glass, silicon dioxide, modified urethane dimethacrylate resin, polymerizable dimethacrylate resin, polymerizable trimethacrylate resin, triethyleneglycol dimethacrylate, camphorquinone (CQ) photoinitiator, photoaccelerator, butylated hydroxyl toluene (BHT), UV stabilizer, titanium dioxide, inorganic iron oxide, fluorescing agent	Low (flowable)	47.3 vol%	20–10 µm	Prime & Bond Active (Dentsply DeTrey)	Phosphoric-acid–modified acrylate resin, multifunctional acrylate, bifunctional acrylate, acidic acrylate, isopropanol, water, initiator, stabilizer
2	Tetric PowerFill (TPF, Ivoclar Vivadent; Schaan, Liechtenstein	IVA	Dimethacrylates, barium glass, ytterbium trifluoride, mixed oxide, copolymers, additives, initiators, stabilizers, pigments	High (moldable)	76-77 wt% or 53-54 vol%	40 nm–3 µm	Adhese Universal (Ivoclar Vivadent)	Methacrylates, ethanol and water, silicon dioxide, initiators and stabilizers
3	Venus Bulk Fill (VBF, Heraeus Kulzer; Hanau, Germany)	Universal shade	Methacrylate monomers (UDMA, EBADMA), barium-alumino-fluoro-borosilicate glass, ytterbium trifluoride glass, silicon dioxide	Low (flowable)	65 wt% or 41 vol%	0.02 μm–5 μm	iBOND Universal (Heraeus Kulzer)	Acetone/water-based solution of light-activated methacrylate monomers
4	3M Filtek One Bulk Fill (FOB, 3M Oral Care; St Paul, MN, USA)	A2	Aromatic urethane dimethacrylate (AUDMA), additional-fragmentation monomers (AFM), 1,12-dodecanediol dimethacrylate (DDMA), urethane dimethacrylate (UDMA), ytterbium fluoride particles (YbF3), silicon oxide filler, zirconium oxide filler, zirconium oxide/silicon dioxide cluster	High (moldable, but comparable to a flowable composite resin)	76.5 wt% or 58.4 vol%	4 nm–100 nm	3M Scotchbond Universal (3M Oral Care)	MDP phosphate monomer, dimethacrylate resins, Vitrebond copolymer, filler, ethanol, water, initiators, silane
5	SonicFill 3 (SF3, KaVo Kerr, KaVo Dental, Biberach, Germany)	A2	Chemically infused mixed oxides, barium glass filler, silicon dioxide, ytterbium trifluoride, bis-EMA, bis-GMA and TEG-DMA resins	Low (through sonic activation)	81 wt% or 65.9 vol%	40 nm–10 μm	OptiBond eXTRa Universal (KaVo Dental)	Ternary solvent system (water/ethanol/acetone), 2-hydroxyethyl methacrylate, glycerol dimethacrylate, glycerol phosphate dimethacrylate, trimethylolpropane trimethacrylate, sodium hexafluorosilicate
6	VisCalor bulk (VCB, Voco; Cuxhaven, Germany)	A2	Dimethacrylate, camphorquinone, BHT, amines, SiO2 nanoparticles, glass-ceramic (Ba-Al-Silikat), pigments: iron oxide and titanium dioxide	Low (through temperature rise; thermo-viscous)	83 wt%	SiO2 nanoparticles 20–40 nm Average particle size of glass-ceramic (barium aluminium silicate): 1.2 µm	Futurabond U (Voco)	Liquid 1: dimethacrylates (bis-GMA, HDDMA, UDMA, HEMA), silicon dioxide, acid modified methacrylate (10 MDP), camphorquinone, BHT, amines Liquid 2: ethanol, water, DC catalyst
7	everX Flow (EXF, GC Germany; Bad Homburg von der Höhe, Germany)	Bulk shade	E-glass fibers, barium glass, bis-MEPP, TEG-DMA, UDMA	Low (flowable)	70 wt% (25 wt% glass fibers and 45 wt% particulate fillers)	Average size particle filler: 700 nm; average length of glass fibers: 140 µm; average diameter of glass fibers: 6 µm	G-Premio Bond (GC Europe)	4-MET, MDP, thiophosphate ester monomer (MDTP), dimethacrylate monomers, distilled water, acetone, silicon dioxide, photoinitiator
8	Surefil One (SFO, Dentsply DeTrey)	A2	Aluminium phosphorus strontium sodium fluorosilicate glass, water, highly dispersed silicon dioxide, acrylic acid, polycarboxylic acid, ytterbium fluoride, bifunctional acrylate, self-curing initiator, 4-tert-butyl-N,N-dimethylaniline, iron oxide pigments, barium sulphate pigment, manganese pigment, camphorquinone (photoinitiator), stabilizer	High (moldable)	74 wt%	circa 2.0 µm		
9	Dyract eXtra (DEX, Dentsply DeTrey)	A2	Urethane dimethacrylate (UDMA), carboxylic acid modified dimethacrylate (TCB resin), triethylene glycol dimethacrylate (TEG-DMA), trimethacrylate resin (TMP-TMA), dimethacrylate resins, camphorquinone, ethyl-4(dimethylamino)benzoate, butylated hydroxytoluene (BHT), UV stabilizer, strontium alumino-sodium flluorophosphorosilicate glass, highly dispersed silicon dioxide, strontium fluoride, iron oxide and titanium dioxide pigments	High (moldable)	77 wt%	circa 0.9 µm	Prime & Bond NT (Dentsply DeTrey)	Urethane dimethacrylate (UDMA), trimethacrylate, phosphoric acid modified acrylate resin (PENTA), highly dispersed silicon dioxide, camphorquinone photoinitiator, ethyl 4(dimethylamino)benzoate, butylated hydroxytoluene (BHT), cetylamine hydrofluoride, acetone

Specimens were stored in distilled water in an incubator at 37°C (Typ B20 Heraeus Kulzer; Hanau, Germany) for 28 days (T_1_). Thermocycling (TCS 30, Syndicad; München, Germany) for 2500 cycles at 5°C/55°C (T_2_) and chewing simulation for 100,000 cycles (antagonist steatite: 6 mm diameter, 50 N, 1.67Hz; T_3_) followed separately.

After T_1_, T_2_, and T_3_, silicone impressions were taken and cast with AlphaDie MF (Schütz Dental; Rosbach, Germany). All replicas were then affixed to specimen holders. Replicas for scanning electron microscopy (SEM) (T_1_ and T_3_) were sputter-coated with gold (POLARON SC502 sputter-coater, Fisons plc; Ipswich, UK), and replicas for wear analysis (T_2_ and T_3_) were coated with Cerec Optispray (Sirona Dental Systems; Bensheim, Germany).

For quantitative marginal analysis, SEM images (Amray 1810, Amray; Bedford, MA, USA; 10 kV accelerating voltage; 200X magnification) of proximal margins were taken, merged, and analyzed with “Fiji is Just Image J” Freeware (Wayne Rasband, National Institutes of Health; Bethesda, MD, USA) in combination with “KHK’s Quantigap” (Free-ware, KHK). For SEM examination, margin sections were classified according to eight different criteria: “perfect margin”, “overhang”, “positive step formation”, “negative step formation”, “gap”, “non-judgeable”, “fracture”, and “paramarginal gap”.

For wear analysis, occlusal faces of the coated replicas were scanned with the structured-light scanner ATOS Core 45 (measuring volume: 45 x 30 x 25 mm; working distance: 170 mm; point spacing: 0.02 mm) in combination with the ATOS Prof 2018 software (both from GOM; Braunschweig, Germany). After the superimposition of the scan bodies before and after TML, the height loss in the occlusal contact area was evaluated as maximum, minimum and mean wear with the software program GOM Inspect 2020 (GOM).

The non-parametric Kruskal-Wallis test, combined with the Bonferroni-Holm correction, was used to compare outcomes of quantitative margin analysis and wear analysis among the groups and the Wilcoxon signed-rank test was used to examine the development of marginal outcomes. A p-value < 0.05 was considered statistically significant. The results were presented as medians and interquartile ranges (median [IQR]).

## Results

**Fig 1:**
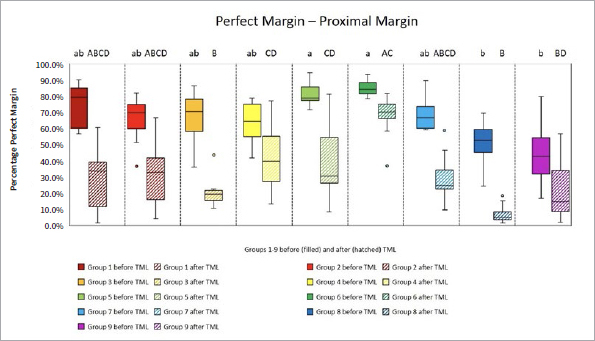
Box plots showing results of margin analysis for the criterion “perfect margin” of each group before (filled) and after (hatched) TML. Results are presented as medians (central lines), IQRs (boxes), and whiskers (minimum/maximum), outliers (circles) are values being below/above 1.5x the interquartile range. Different capital letters: significant differences between groups; different lowercase letters: significant differences between groups; Kruskal-Wallis test, p < 0.05.

**Fig 2:**
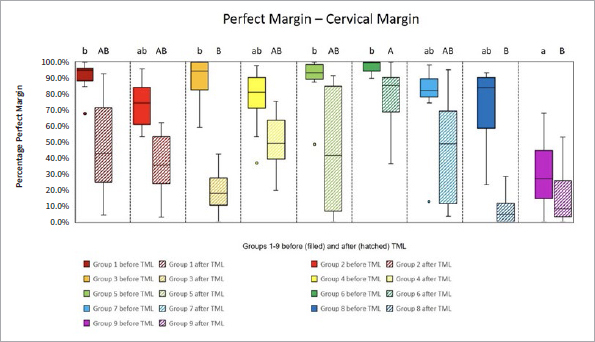
Box plots showing results of margin analysis for the criterion “perfect margin” of each group before (filled) and after (hatched) TML. Results are presented as medians (central lines), IQRs (boxes), and whiskers (minimum/maximum), outliers (circles) are values being below/above 1.5x the interquartile range. Different capital letters: significant differences between groups; different lowercase letters: significant differences between groups; Kruskal-Wallis test, p < 0.05.

**Fig 3:**
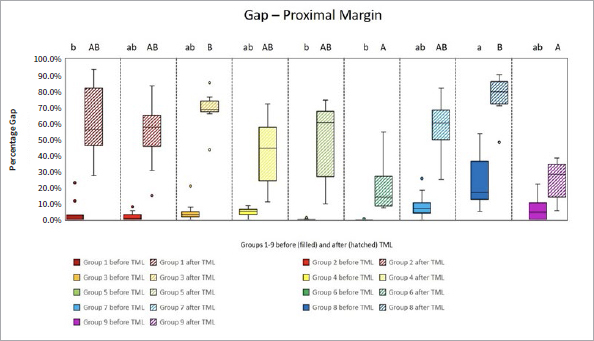
Box plots showing results of margin analysis for the criterion “gap” of each group before (filled) and after (hatched) TML. Results are presented as medians (central lines), IQRs (boxes), and whiskers (minimum/maximum), outliers (circles) are values being below/above 1.5x the interquartile range. Different capital letters: significant differences between groups; different lowercase letters: significant differences between groups; Kruskal-Wallis test, p < 0.05.

**Fig 4:**
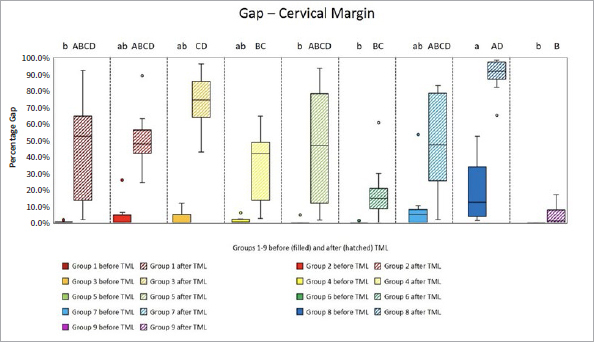
Box plots showing results of margin analysis for the criterion “gap” of each group before (filled) and after (hatched) TML. Results are presented as medians (central lines), IQRs (boxes), and whiskers (minimum/maximum), outliers (circles) are values being below/above 1.5x the interquartile range. Different capital letters: significant differences between groups; different lowercase letters: significant differences between groups; Kruskal-Wallis test, p < 0.05.

**Fig 5:**
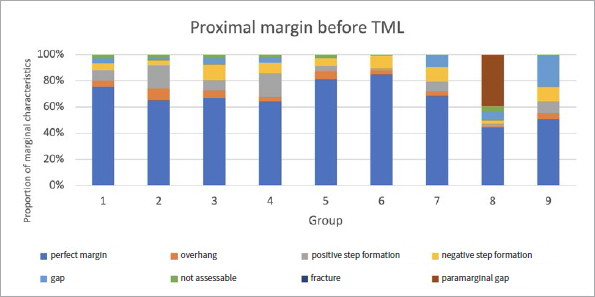
Different marginal quality criteria at proximal margins before TML.

**Fig 6:**
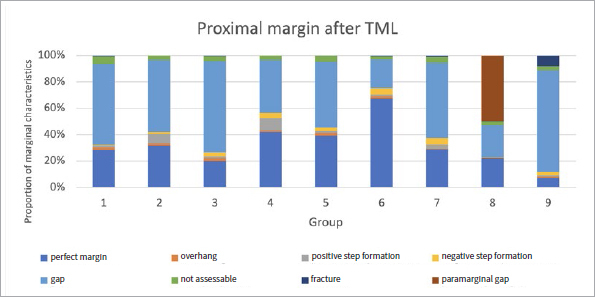
Different marginal quality criteria at proximal margins afer TML.

**Fig 7:**
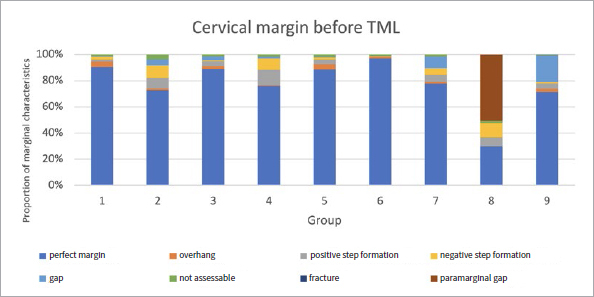
Different marginal quality criteria at cervical margins before TML.

**Fig 8:**
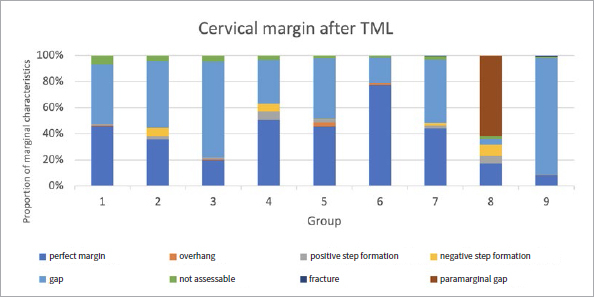
Different marginal quality criteria at cervical margins after TML.

The proportions of the different criteria for marginal analysis of proximal and cervical margins before and after TML are presented in Figs 1 to 8. A significant reduction in perfect margins was observed on proximal and cervical margins for all groups after TML (p < 0.02), whereas marginal gap formation increased for all groups (p < 0.02). The highest proportions of perfect margins after TML were recorded for group 6 (75% [64–82%]), a thermoviscous BFC, while the lowest proportion of perfect margins after TML was seen in group 8 (7% [2%–13%]), the self-adhesive composite hybrid. In contrast, group 8 demonstrated the highest values of marginal gaps before (22% [8%–33%]) and after (84% [78%–90%]) TML, while group 6 showed the lowest values of marginal gaps before (0% [0%]) and after (13% [7%–33%]) TML. Differences between groups before and after TML are presented in Figs 3 and 4 for the criteria “perfect margin” and “gap”. Wear analysis demonstrated no significant differences between the groups (p > 0.05). The results are presented in Table 3.

**Table 3:** Results of wear analysis

Material	Median (IQR) in µm		
	Minimum distance	Maximum distance	Mean distance
Group 1	-80.268 (-113.488 to -69.506)	-134.715 (-173.357 - -108.462)	-115.084 (-142.965 to -97.006)
Group 2	-35.984 (-64.186 to -31.165)	-62.239 (-122.402 to -50.520)	-51.550 (-105.364 to -40.608)
Group 3	-40.626 (-54.316 to -31.645)	-60.579 (-105.088 to -39.380)	-52.660 (-84.967 to -36.258)
Group 4	-42.771 (-53.045 to -32.778)	-73.902 (-85.924 to -62.870)	-62.742 (-67.558 to -51.709)
Group 5	-64.631 (-78.205 to -38.543)	-97.895 (-162.502 to -64.652)	-81.787 (-128.933 to -56.934)
Group 6	-41.759 (-59.625 to -21.716)	-79.201 (-170.519 to -59.243)	-66.855 (-120.671 to -48.077)
Group 7	-39.089 (-48.324 to -32.249)	-83.792 (-111.444 to -56.356)	-63.246 (-86.725 to -48.918)
Group 8	-41.290 (-50.048 to -38.338)	-127.991 (-268.182 to -115.059)	-83.077 (-117.438 to -77.222)
Group 9	-39.080 (-42.738 to -30.645)	-132.967 (-174.250 to -101.646)	-93.843 (-123.772 to -73.474)

Kruskal-Wallis test (Bonferroni-Holm correction): no significant differences between groups (p > 0.05).

## Discussion

The basic principle of quantitative marginal analysis via the replica technique and SEM was already described in 1989 by Roulet et al.^37^ It is a complex, time-consuming procedure^16^ and is considered the gold-standard method for evaluating the marginal integrity of restorations.^17^ Furthermore, this technique can be used for laboratory studies as well as clinical trials.^16^ One of the disadvantages of this technique is that the assessment of a three-dimensional surface is based on two-dimensional images.^21^ Even if marginal gaps are detected, it is not possible to determine their true depth. Therefore, a prediction of bacterial penetration into the interface is not possible. Moreover, there is no clear consensus on whether a patent marginal opening necessarily results in the development of secondary caries.^29^ According to Pashley,^33^ there is only a weak correlation between the extent of microleakage detected in vitro and the clinical success of a restorative material. However, if a material does not show microleakage in vitro, there is a higher probability of clinical success for the material.^16,33^

Polymerization of composite resins causes molecular crowding of the resin matrix, which results in volumetric shrinkage.^9^ Usually, this polymerization shrinkage is on the order of 1.5%–5% by volume.^13^ Depending on the amount of polymerization stress at the margin, adhesives must provide a certain ability to counteract shrinkage and therefore help prevent marginal gaps by bonding the filling material to the cavity walls.^9^

In the present study, different universal adhesives were used in combination with their corresponding filling materials, plus one self-adhesive material (no adhesive required). Combining BFCs and adhesives of the same manufacturers guarantees maximum compatibility, although it could have led to variations in marginal quality. Nevertheless, using restorative systems of the same manufacturer presents the opportunity to investigate the combinations in their original form as recommended by the manufacturers. When shrinkage-prone materials are successfully bonded, however, substantial stress arises within the material.^9^ This can affect and fatigue the enamel/dentin bond, promoting gaps and microcracks in or near the interface.^9,13^ Due to its higher polymerization shrinkage, it was obvious that the tested compomer (Dyract eXtra, DEX, group 9) was the only filling material that showed paramarginal fractures/gaps, ie, located in the enamel next to the interface between the restoration and the tooth. These paramarginal gaps often spread over extensive, continuous lengths, detected even before TML, but increase further after TML. These effects could be explained by higher polymerization shrinkage on the one hand and a strong bond on the other.

A higher C-factor – that is, a higher ratio of bonded to unbonded surfaces – of a restoration causes more polymerization shrinkage stress within the restorative material. Class-II cavities normally possess a C-factor of 1–2, depending on application technique.^12^ An application in several layers can reduce the C-factor for every layer used, and each subsequent layer can fill the lost space due to polymerization shrinkage of the previous layer. Gupta et al^19^ demonstrated that an oblique-incremental application technique, as used for the compomer group in the present study, can significantly reduce microleakage at the margins of compomer fillings in primary molars in vitro, compared to bulk-fill application. However, none of the tested placement techniques could completely prevent microleakage.^19^ Despite omitting a meticulous layering technique, BFCs exhibit lower polymerization shrinkage^5^ and lower shrinkage stress^6^ in vitro. BFCs are usually characterized by increased translucency, which can be achieved by reducing filler content and increasing the size of the filler particles.^22^ Due to the resulting smaller interface between fillers and resin matrix, the light from the polymerization unit entering the material scatters more, which results in higher curing depths.^23^ Flexural and fracture strength of BFC as well as their marginal quality are similar to those of CRs in vitro.^6^

In the present study, the thermo-viscous BFC VCB (VisCalor bulk) showed the best results with respect to marginal integrity. The viscosity change based on temperature changes seems to be an advantage of this particular material. The improved marginal adaptation could be explained due to increased wettability when the BFC is preheated. After the application of the material, VCB cools down and its viscosity increases, which allows modelling of restoration surfaces. In terms of handling, it is important to bear in mind that – once the material has been warmed up – the filling procedure must be performed within 150 s. Otherwise, a new capsule has to be used, which takes more time and thus could be disadvantageous in pediatric dentistry.

In the present study, the self-adhesive bulk-fill material SFO (Surefil One), the composition of which closely resembles that of a resin-modified glass-ionomer (GI), performed worst in terms of marginal adaptation. In addition, retention and marginal adaptation of GI restorations proved to be worse than those of BFC and CR (composite resin) restorations in primary molars in vivo.^2^ Conversely, SFO provided comparable marginal adaptation to CRs in combination with self-etch adhesives in permanent molars in another in-vitro study.^14^ Regarding shear bond strength, SFO provided better results in permanent molars in vitro than other self-adhesive restorative materials, but poorer results than a CR in combination with a universal adhesive.^24^ Latta and Radniecki^24^ demonstrated that the smear layer affects the shear bond strength of SFO, but not when using a universal adhesive in self-etch mode in combination with a conventional composite resin (CR). In terms of fracture strength and fracture toughness, SFO is ranked between the CRs, which perform better, and the GIs, which perform worse.^25^ This is supported by the present study, where SFO clearly showed the highest proportion of fractures in marginal areas compared to the other adhesive materials after TML. Except for SFO, VBF (Venus Bulk Fill) exhibited the highest marginal gap rates, as also confirmed in an in-vitro study by Benetti et al,^5^ in which VBF showed significantly more marginal gaps than did a CR. Paganini et al^32^ also found that VBF provided the worst marginal integrity in vitro, which was even worse than the integrity of a CR applied in bulk-fill technique with an incremental thickness of 4 mm. In addition, VBF showed the highest polymerization shrinkage compared to other BFCs/CRs in several laboratory studies,^5,40^ which could be one reason for the higher levels of marginal gaps in the VBF group. Conversely, a clinical trial in a split-mouth design by Ehlers et al^10^ investigated VBF in comparison to DEX for the restoration of Class-II cavities in primary molars. The follow-up period was one year. Esthetic as well as functional and biological properties of the restorations were investigated. Almost all restorations were evaluated after one year, all restored teeth were vital, lacked postoperative symptoms, and were caries free. The bulk-fill materials showed a good performance, comparable to that of the compomer. It was inferior to the compomer only in terms of esthetics, due to its increased translucency and being available in only one universal shade.^10^ These findings lead to the assumption that, if microscopic paramarginal gaps for DEX (Dyract eXtra) and a high rate of marginal gaps for VBF also occur in vivo, neither of them seems to have any influence on the success of the restorations within one year of clinical service. Akman and Tosun^2^ compared one high viscosity and one sonically activated BFC with a GI and a CR used for Class-II restorations of primary molars in vivo. Both of the BFCs and the CR showed good results, comparable to each other after one year of follow-up. The GI performed worse than the other tested materials in terms of retention and marginal adaptation.^2^ The sonically activated material SonicFill 3 also showed superior results in terms of marginal integrity before TML in the present study. The improved initial marginal adaptation may be explained by the reduction of viscosity during sonically activated application. A clinical study in a split-mouth design by Öter et al^31^ examined a BFC and a CR for restoring Class-I cavities in primary molars. After one year, the BFC provided results comparable to those of the CR, but it was noted that more cases of postoperative hypersensitivity occurred at baseline with the BFC. However, during the restoration procedure, indirect capping of the pulp was indicated several times due to the extensive depth of the defects. In addition, the BFC showed a significant reduction in marginal integrity in terms of clinically detectable gap formation during the observation period, but there were no significant differences between the materials tested at different points in time.^31^ Sarapultseva and Sarapultsev^38^ investigated a flowable BFC (SDR flow+, Table 2) and a nano-ceramic CR used for Class-I restorations. After two years of follow-up, both restorative materials provided clinically acceptable and almost identical results. None of the restorations exhibited secondary caries or resulted in postoperative symptoms.^38^

Due to the fact that restorations of primary teeth usually have to last only five to six years,^36^ wear resistance does not play a major role. This is also the reason why a relatively low number of chewing simulation cycles were used, in keeping with previous laboratory studies testing restorations of primary teeth.^28^ Furthermore, wear resistance of primary-tooth enamel is lower than permanent-tooth enamel.^18^ This is the reason why no capping layer was used for bulk-fill materials in the present study, contrary to the recommendations of some manufacturers. In terms of wear resistance, compomers range between CRs and the GIs.^18^ According to the present findings, neither the BFCs nor SFO differed from compomers with regard to wear resistance. Engelhardt et al^42^ found a correlation between the microhardness and the relative wear of a restorative material.^11^ BFC^6^ as well as compomers^42^ exhibit lower microhardness values in vitro than do CRs, which could explain the similar results with respect to wear resistance of the materials tested in this study. In another in-vitro study, SFO demonstrated significantly worse wear resistance than amalgam and a CR, but better wear resistance than other self-adhesive materials, such as GIs, in permanent molars after TML.^14^ Considering its comparable wear resistance to the BFCs and the compomer, as well as the fact that SFO is a dual-curing material, SFO might be an option as a better interim restoration for less compliant patients.

## Conclusion

The results of the present study demonstrated significant differences among the materials tested in terms of marginal integrity, but not with respect to wear resistance. In summary, viscosity-changing bulk-fill materials seem to be a promising option in terms of marginal adaptation, but further long-term clinical trials are needed to evaluate the success of bulk-fill materials in primary molars. The null hypothesis had to be rejected.

## Clinical relevance

Bulk-fill composites offer an effective means of restoring Class-II cavities in primary molars.
